# Who loses more? Identifying the relationship between hospitalization and income loss: prediction of hospitalization duration and differences of gender and employment status

**DOI:** 10.1186/s12889-022-12647-6

**Published:** 2022-02-04

**Authors:** Minsung Sohn, Daseul Moon, Patricia O’Campo, Carles Muntaner, Haejoo Chung

**Affiliations:** 1 Division of Health and Medical Sciences , The Cyber University of Korea, Seoul, Republic of Korea; 2 BK21PLUS Program in Public Health Sciences, Korea University, Seoul, Republic of Korea; 3 Centre for Urban Health Solutions, St Michael’s Hospital, Toronto, Canada; 4 Dalla Lana School of Public Health, University of Toronto, Toronto, Canada; 5 Bloomberg School of Nursing, University of Toronto, Toronto, Canada; 6 BK21FOUR R&E Center for Learning Health Systems, Korea University, Seoul, Republic of Korea; 7 School of Health Policy and Management, Korea University, Seoul, Republic of Korea

**Keywords:** Social protection, Sickness benefits, Hospitalization, Income loss, Workers, Employment arrangement

## Abstract

**Background:**

The major determinants of health and well-being include wider socio-economic and political responses to poverty alleviation. To data, however, South Korea has no related social protection policies to replace income loss or prevent non-preferable health conditions for workers. In particular, there are several differences in social protection policies by gender or occupational groups. This study aimed to investigate how hospitalization affects income loss among workers in South Korea.

**Methods:**

The study sample included 4876 Korean workers who responded to the Korean Welfare Panel Study (KoWePS) for all eight years from 2009 to 2016. We conducted a receiver operating characteristics (ROC) analysis to determine the cut-off point for the length of hospitalization that corresponded to the greatest loss of income. We used panel multi-linear regression to examine the relationship between hospitalization and income loss by gender and employment arrangement.

**Results:**

The greatest income loss for women in non-standard employment and self-employed men was observed when the length of hospitalization was seven days or less. When they were hospitalized for more than 14 days, income loss also occurred among men in non-standard employment. In addition, when workers were hospitalized for more than 14 days, the impact of the loss of income was felt into the subsequent year.

**Conclusion:**

Non-standard and self-employed workers, and even female standard workers, are typically excluded from public insurance coverage in South Korea, and social security is insufficient when they are injured. To protect workers from the vicious circle of the poverty-health trap, national social protections such as sickness benefits are needed.

**Supplementary Information:**

The online version contains supplementary material available at 10.1186/s12889-022-12647-6.

## Introduction

The World Health Organization (WHO) has proposed universal health coverage (UHC) as a strategy for national health policies that “all people and communities can use the promotive, preventive, curative, rehabilitative, and palliative health services they need, of sufficient quality to be effective, while also ensuring that the use of these services does not expose the user to financial hardship [1].” Given this definition, the WHO’s UHC considers catastrophic health expenditures as a determinant of household income insecurity. Despite the global attention that South Korea has received for its achievement in rapidly forming a universal health insurance system in 12 years, the system requires only about 65% of copayment which cause higher level of out-of-pocket healthcare expenditure.

In addition, financial hardship due to sudden and unpredictable illness can be caused by not only direct medical costs (i.e., catastrophic health expenditure), but also indirect costs such as loss of earned income and transportation fees associated with the use of medical services [2]. Those in poor health have a lower chance of achieving favorable levels of income. Employers may decide to cut employees’ wages, demote them to lower positions, and dismiss or replace them to offset costs incurred by employees’ lower productivity and/or absenteeism. Poverty is a significant social determinant of ill health. The absence of social health protection to guarantee workers’ optimum level of income for living when they are sick (i.e., paid sick leave, sickness benefit, etc.) entraps them into a vicious cycle of poverty and poor health.

In this regard, sickness benefits or paid sick leave have been introduced to ensure both leave from work and cash benefits to replace wage loss during workers episodes of illness. While sickness benefits or paid sick leave are instrumental in protecting workers’ and their families health and economic status [3], South Korea is one of the few countries that do not require employers to provide leave to employees for non-work-related illness. Furthermore, only about 7 % of enterprises provide paid sick leave to their workers [4]. Recently, Korean public policy has been making efforts to mitigate the blind spot of social coverage resulting from a sudden change in employment relations. In particular, the COVID-19 pandemic has prompted active discussions on the introduction of sickness benefits in South Korea. Therefore, it would be timely to generate empirical evidence on the effectiveness of sickness benefits to inform the design and introduction of a nationwide system.

Previous studies have shown an association between health shocks and income loss. Most studies have examined income loss or decline among individuals diagnosed with cancer [5–9]. Other studies have operationalized health shock as diabetes [10], health satisfaction [11], or hospital admission [12, 13]. This evidence supports the notion that health shocks are associated with income loss. Three studies conducted in South Korea have also shown that health shocks decrease income [14–16]. All three studies have defined health shock differently: health expenditures in relation to earned income [14], having serious diseases or not [15], and hospitalization longer than three days [16]. Higher out-of-pocket expenses for using medical services and medication costs compared to earned income were associated with decreased total income, including wages, private and public transfers, and asset income [14]. When cancer, a cardiac disorder, or cerebrovascular disease occurs, workers’ income is substantially reduced (33.7 % for cancer, 29.3 % for cardiac disorder, and 45.1 % for cerebrovascular disease) [15]. Workers who experienced hospitalizations longer than three days in the previous two years earned 23.6 % less than workers in comparable positions who had not [16].

However, the empirical question about the degree to which health can influence income for different groups based on their differential exposure to social determinants (e.g., employment and occupational status, gender) remains unclear [17]. It is necessary to identify the different patterns of causal relationships between health shock and income loss, stratified by employment arrangements and gender, while simultaneously considering the dual and gendered labor market of South Korea. In South Korea, the proportion of non-standard workers accounts for nearly 32.9 % (6.5 %) of the entire workforce, twice higher than the number in other Organization for Economic Co-operation and Development (OECD) member countries. One-quarter of Korean women are employed in low-paying, non-standard positions. The wage gap between Korean women and men is 32.5 %, ranked first among the OECD countries [18]. Moreover, if better health protects against income loss, then a more unequal distribution of health between different social groups should lead to larger income disparities. Indeed, total family income declined by up to 4.8 % among men and 85 % among women when they are diagnosed with cancer [5].

One study examined whether the association between health shock and income loss differed by employment arrangement and gender [16]. The study concluded that income loss due to hospitalization was more pronounced among non-standard workers than standard workers, and unemployment due to hospitalization was more pronounced among women than among men. The present study goes a step further to identify the influence of intersects between employment arrangements and gender on income loss. The aim of this study is to identify the causal relationship between health shock and income loss and different patterns of associations by workers’ employment arrangements and gender.

## Materials and Methods

### Data and Study population

We used data from the latest six waves of the Korean Welfare Panel Study (KoWePS) (2009–2016). The KoWePS is the largest sampling data and nationally representative longitudinal study in South Korea. It has surveyed “the dynamic aspects and varying needs of people over the course of their lives, including living conditions, socio-economic status (SES), and health status,” since 2006.

**Fig. 1:**
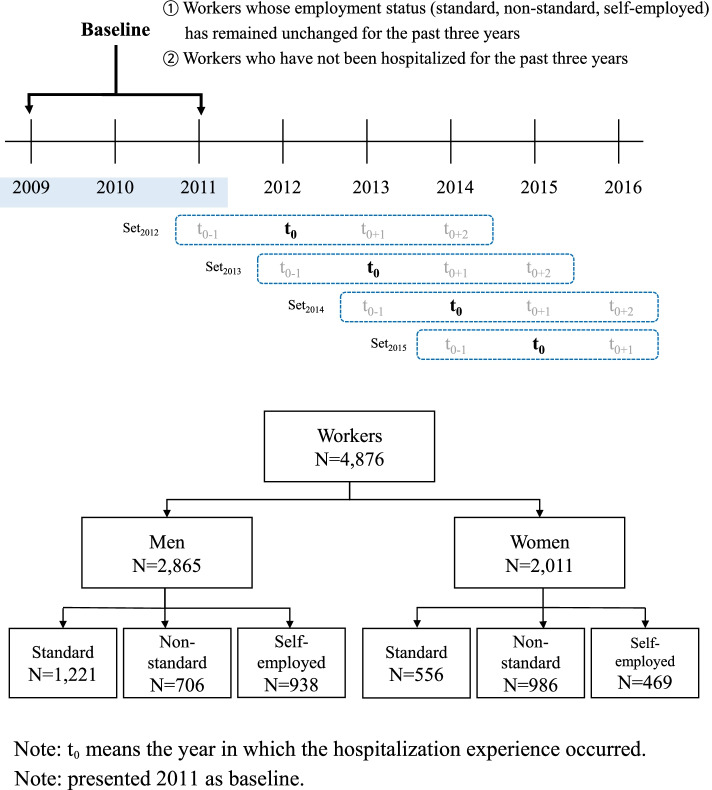
Flow of sample selection in this study

Figure 1 shows the selection process for the study population. In the first step, baseline data from 2009 to 2011 was gathered. Baseline data was established as follows: a) only workers whose employment status remained unchanged for three years were selected, and b) those who had other types of contractual works, such as workfare, employer, unpaid family workers, non-standard work arrangements, and the self-employed were excluded. Finally, workers who had never been hospitalized in the previous three years were selected, to ensure a sample of healthy workers. Further, the data until 2016 was merged with the baseline data. Next, a) the data with missing values in the major variables was excluded in the analysis, and b) only those who responded for all eight years from 2009 to 2016 were selected, to establish balanced panel data (*n*=4876 each year).

### Variables

#### Dependent variable: Change of income

The dependent variable in this study is the change in an individual's earned income. Earned income changes were measured by subtracting the average annual income for the three baseline years from the earned income for the next five years (2012 to 2016). The average earned income of the baseline years was used to minimize or control the effects of economic growth and/or inflation. In the case of self-employed agriculture, forestry, farming, or fishing workers, negative earned income values may occur because of the possibility of a net loss. In other words, income (profit) may be negative because the total costs may be higher than total sales.

#### Independent variable: Hospitalization

Health shocks were conceptualized as experience of hospitalization and dichotomized hospitalization to “Yes” and “No”. To dichotomize the variable, the length of hospitalizations leading to a substantial loss of income were identified by analyzing a receiver operating characteristics (ROC) curve. The ROC curve predicted that hospitalizations for three days would lead to a substantial income loss. Therefore, “Yes” was defined as having an experience of hospitalization lasting for greater than three days. “No” was defined as having experience of hospitalization of less than three days, or having no experience of hospitalization. Additionally, 7 days and 14 days were used to dichotomize the variables to identify differences in disease severity. Further, individuals who reported their reasons for hospitalization as childbirth, medical checkups, and convalescence were excluded because such hospitalizations were not caused by sudden health problems.

#### Groups: employment arrangement and gender

To examine whether the relationship differs by social groups, workers were stratified based on employment arrangement and gender. Employment arrangement was based on contractual types (i.e., permanent, temporary, daily, and self-employed), working hours (full-time and part-time), and status of occupation and employment insurance. Employment arrangements were categorized into standard employment, non-standard employment, and self-employment. Individuals were defined as having standard work arrangements if they satisfied the following three conditions: permanent contract, full-time, and having both occupation and employment insurance. Respondents of the independent contractual type were defined as self-employed. To define the small self-employed, five or more self-employed people were excluded in this study. Respondents were defined as having non-standard work arrangements if they had temporary or daily contracts, whether they worked full-time, or had insurance. If respondents had permanent contracts but were part-time workers or did not have both occupation and employment insurance, they were also defined as non-standard employees.

#### Covariates: age, education, marital, public health insurance, private health insurance, poverty, chronic disease, disability, and health status

In this study, socio-demographic characteristics and SES were controlled to identify the net effect of hospitalization on income loss among workers. Age was operationalized into three groups: 18 ≤ age < 45 years, 45 ≤ age < 65 years, and age ≥ 65 years. Older workers were included to reflect the characteristics of the informal labor market in South Korea. Educational attainment was classified into four groups: under elementary school, middle school, high school, and college or higher. Marital status was classified into four categories: married couple, widowed, divorced or separated, and single (never married). Both public and private health insurance were investigated in binary; National Health Insurance (NHI) subscribers and individuals with private medical insurance were used as references. Poverty was defined as those who received the basic living allowance system: yes or no. Chronic disease and disability were also categorized as binary: yes or no. Subjective health status was investigated on a five-point Likert scale, and the reference was for the healthiest individuals. The square root of earned income of the year was also adjusted to control for the effects of the annual increment.

### Statistical analyses

First, the ROC analysis was conducted to determine the cut-off point for the length of hospitalization that results in a great loss of income. Second, the difference in the change of income loss was assessed between those who experienced hospitalization and those who did not. The differences between them were examined by gender and employment arrangements. Third, fixed effect panel regression analysis was used to investigate the relationship between hospitalization and changes in income loss, controlling for other related variables.

Y_*it*_ = β_0_ + β_1_(hospitalization_α_)_*it*_ + β_2_(individual confounders)_*it*_ + u_*i*_ + ε_*it*_

*i*: individual, *t*: year, α: the year of hospitalization, the year of hospitalization+1year, the year of hospitalization+2year, Y: income loss,

- Individual confounders: age, education, marital status, public health insurance, private health insurance, poverty, chronic disease, disability, and subjective health status

- group: gender, employment status

## Results

### Characteristics of samples

The baseline characteristics of the participants in the panel regression analysis from 2011 to 2016 are summarized in Table 1. As of 2011, as the baseline, there was a total sample of 4876 workers each year. Of these, 58.76 % and women were 41.24 %. Those between 45 and 65 years old were the highest in number at 42.76 %, and the mean age of all workers was 55.02±15.16 years. The highest number of workers (34.29 %) graduated from college, followed by high school, under elementary school, and middle school graduates. Regarding marital status, married couples were the most common at 71.37 %. The average annual income is approximately 23±21 million won. At baseline, the employment arrangements, standard, non-standard, and self-employed workers who had not changed their occupations for the past three years were 36.44 %, 34.70 %, and 28.86 %, respectively. The proportion of workers with hospitalizations of more than 3, 7, and 14 days was 7.69 %, 6.03 %, and 3.22 %, respectively.

**Table 1:** Sample Characteristics (*N*=4,876)

	**2011**		**2012**		**2013**		**2014**		**2015**		**2016**	
	***N***	**%**	***N***	**%**	***N***	**%**	***N***	**%**	***N***	**%**	***N***	**%**
Age												
18 ≤ age < 45	1,378	28.26										
45 ≤ age < 65	2,085	42.76										
age ≥ 65	1,413	28.98										
Education												
Under elementary school	1,015	20.82	1,010	20.71	1,010	20.71	1,011	20.73	1,012	20.75	1,010	20.71
Middle school	600	12.31	604	12.39	600	12.31	599	12.28	598	12.26	599	12.28
High school	1,589	32.59	1,582	32.44	1,582	32.44	1,574	32.28	1,570	32.20	1,568	32.16
College or higher	1,672	34.29	1,680	34.45	1,684	34.54	1,692	34.70	1,696	34.78	1,699	34.84
Poverty												
Yes	926	18.99	1,022	20.96	1,079	22.13	1,053	21.60	1,051	21.55	1,089	22.33
No	3,950	81.01	3,854	79.04	3,797	77.87	3,823	78.40	3,825	78.45	3,787	77.67
Marital status												
Married couple	3,480	71.37	3,481	71.39	3,503	71.84	3,512	72.03	3,515	72.09	3,509	71.96
Widowed	393	8.06	413	8.47	422	8.65	439	9.00	460	9.43	487	9.99
Divorced or Separated	282	5.78	290	5.95	299	6.13	311	6.38	323	6.62	325	6.67
Single	721	14.79	692	14.19	652	13.37	614	12.59	578	11.85	555	11.38
Public health insurance												
NHI	4,772	97.87	4,778	97.99	4,761	97.64	4,788	98.20	4,776	97.95	4,782	98.07
Medical aid	104	2.13	98	2.01	115	2.36	88	1.80	100	2.05	94	1.93
Private health insurance												
Yes	3,177	65.16	3,228	66.20	3,224	66.12	3,345	68.60	3,360	68.91	3,410	69.93
No	1,699	34.84	1,648	33.80	1,652	33.88	1,531	31.40	1,516	31.09	1,466	30.07
Chronic disease												
Yes	2,036	41.76	2,173	44.57	2,363	48.46	2,236	45.86	2,395	49.12	2,588	53.08
No	2,840	58.24	2,703	55.43	2,513	51.54	2,640	54.14	2,481	50.88	2,288	46.92
Disability												
Yes	336	6.89	345	7.08	353	7.24	357	7.32	364	7.47	364	7.47
No	4,540	93.11	4,531	92.92	4,523	92.76	4,519	92.68	4,512	92.53	4,512	92.53
Health status												
Very good	821	16.84	658	13.49	655	13.43	615	12.61	581	11.92	493	10.11
Good	2,612	53.57	2,714	55.66	2,529	51.87	2,703	55.43	2,665	54.66	2,626	53.86
Nomal	870	17.84	877	17.99	997	20.45	948	19.44	1,010	20.71	1,070	21.94
Bad	558	11.44	583	11.96	661	13.56	585	12.00	577	11.83	639	13.11
Very bad	15	0.31	44	0.90	34	0.70	25	0.51	43	0.88	48	0.98
^b^Income (10,000 won)	2,280.40	2,194.07	2,260.50	2,214.42	2,313.36	2,600.96	2,300.19	2,402.01	2,386.94	2,571.99	2,473.38	2,750.91
Employment arrangement												
Standard	1,777	36.44	1,724	35.36	1,718	35.23	1,708	35.03	1,680	34.45	1,683	34.52
Non-standard	1,692	34.70	1,376	28.22	1,241	25.45	1,157	23.73	1,159	23.77	1,119	22.95
Self-employed	1,407	28.86	1,317	27.01	1,215	24.92	1,162	23.83	1,121	22.99	1,085	22.25
Unpaid family worker, unemployed, or economic inactivity	-	-	459	9.41	702	14.40	849	17.41	916	18.79	989	20.28
Hospitalization (≥3days)												
Yes	375	7.69	436	8.94	415	8.51	435	8.92	459	9.41	470	9.64
No	4,501	92.31	4,440	91.06	4,461	91.49	4,441	91.08	4,417	90.59	4,406	90.36
Hospitalization (≥7days)												
Yes	294	6.03	352	7.22	331	6.79	332	6.81	339	6.95	359	7.36
No	4,582	93.97	4,524	92.78	4,545	93.21	4,544	93.19	4,537	93.05	4,517	92.64
Hospitalization (≥14days)												
Yes	157	3.22	211	4.33	205	4.20	204	4.18	215	4.41	234	4.80
No	4,719	96.78	4,665	95.67	4,671	95.80	4,672	95.82	4,661	95.59	4,642	95.20

^a^mean±standard deviation and range were 55.02±15.16, from 22 to 96

^b^presented mean and standard deviation

Note: presented the average of income for 3 years from 2009 to 2011 in baseline (2011).

### Change of income loss (t_0_, t_0+1_, t_0+2_) by employment arrangement and gender

**Fig. 2:**
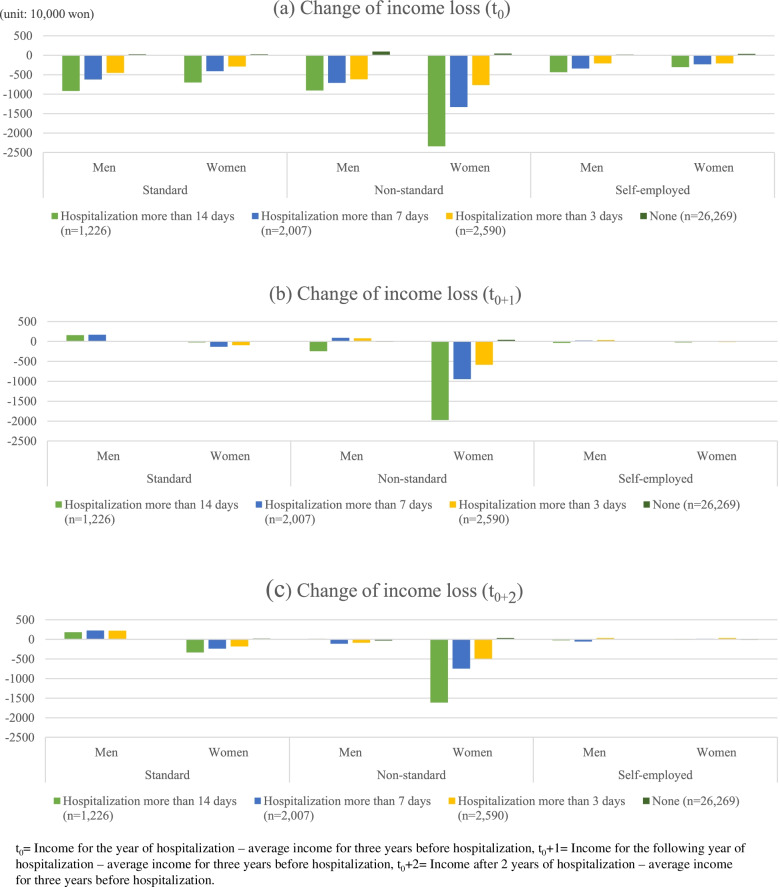
Change of income loss (t_0_, t_0+1_, t_0+2_) by employment arrangement and gender

Figure 2 shows the change in income loss over the previous year according to employment arrangement and gender. In the standard group, both males and females, regardless of whether they were admitted to the hospital, increased their earnings over the previous year. However, the income for the non-standard and self-employed groups decreased compared to the previous year. In particular, those who had more than two days of hospitalization had a significantly higher rate of income decline than those who had one day of hospitalization or were not hospitalized. Furthermore, the decrease was greater among women than among men.

### Relationship between hospitalization (3, 7 and 14 days) and income loss (t_0_, t_0+1_, t_0+2_) by employment arrangement and gender

Table 2 shows the results of the panel regression analysis from 2011 to 2016 to determine the effect of hospitalization experience on income loss according to employment arrangement and gender. Men with standard employment did not experience significant income loss from hospitalization. However, standard and non-standard working women who were hospitalized for more than three days had a greater loss of income than those who were not hospitalized (at all or more than 3 days, standard (t_0_:) β=-439.71, *p*=.038; non-standard (t_0_:) β=-151.67, *p*=.016). Those who were hospitalized long-term suffered greater losses of income than those who were not among non-standard and self-employed men (more than 7 days, non-standard (t_0_:) β=-262.67, p=.048; more than 14 days, non-standard (t_0_:) β=-692.18, *p* < .001; non-standard (t_**0+**1_:) β=-449.29, *p *= .018; self-employed (t_0_:) β=-332.58, *p* = .034), as well as among women (more than 7 days, standard (t_0_:) β=-530.55, *p* = .037; non-standard (t_0_:) β=-220.37, *p* = .002; more than 14 days, non-standard (t_0_:) β=-275.79, *p* = .003; self-employed (t_0_:) β=-190.70, *p* = .046).

**Table 2:** The effect of hospitalization on earned income loss by gender among Korean workers, 2011-2016

	Men									Women								
	Standard (*N*=1,221)			Non-standard (*N*=706)			Self-employed (*N*=938)			Standard (*N*=556)			Non-standard (*N*=986)			Self-employed (*N*=469)		
	β	SE	*p*	β	SE	*p*	β	SE	*p*	β	SE	*p*	β	SE	*p*	β	SE	*p*
Hospitalization (≥3days)																		
t_0_	-156.92	184.64	0.395	-64.72	119.65	0.589	**-305.84**	**120.90**	**0.011**	-102.37	211.73	0.629	**-206.40**	**61.77**	**0.001**	-84.83	76.15	0.265
t_0+1_	138.10	132.14	0.296	-134.06	124.94	0.353	-201.82	133.27	0.130	43.82	233.40	0.851	-62.68	68.62	0.361	-53.52	85.74	0.532
t_0+2_	133.74	130.30	0.305	-54.20	207.76	0.794	-110.23	132.54	0.406	-38.90	235.10	0.869	-64.56	68.23	0.344	11.89	85.17	0.889
Hospitalization (≥7days)																		
t_0_	-80.56	223.81	0.719	-59.91	137.31	0.663	**-317.96**	**131.03**	**0.015**	-11.15	255.18	0.965	**-271.90**	**69.84**	**<.001**	-84.75	81.93	0.301
t_0+1_	167.94	151.16	0.267	-86.96	248.55	0.726	-187.78	144.21	0.193	237.38	280.21	0.397	-51.73	77.79	0.506	2.61	92.56	0.977
t_0+2_	182.58	148.91	0.220	46.41	251.69	0.854	-68.48	143.65	0.634	164.56	281.86	0.559	-62.93	77.32	0.416	57.20	91.76	0.533
Hospitalization(≥14days)																		
t_0_	-312.17	313.98	0.320	-266.21	166.32	0.109	**-543.15**	**160.87**	**0.001**	-244.90	383.54	0.523	**-320.11**	**91.07**	**<.001**	-72.05	98.03	0.462
t_0+1_	156.22	189.33	0.409	**-795.99**	**347.57**	**0.022**	**-345.44**	**175.68**	**0.049**	245.74	422.17	0.561	**-137.61**	**60.18**	**0.043**	-39.12	107.84	0.717
t_0+2_	310.80	186.68	0.096	-399.10	351.98	0.257	-114.45	175.30	0.514	-47.95	425.21	0.910	-68.82	99.95	0.491	125.88	108.46	0.246

Adjusted for age, education, marital status, public health insurance, private health insurance, poverty, chronic disease, disability, and subjective health status

t_0_= Income for the year of hospitalization – average income for three years before hospitalization,

t_0+1_= Income for the following year of hospitalization – average income for three years before hospitalization,

t_0+2_= Income after 2 years of hospitalization – average income for three years before hospitalization.

## Discussion

The UHC suggests that the entire population can use the necessary health services and simultaneously ensure that they are free from financial burdens [19]. Importantly, for UHC to secure universalism and provide high-quality services, it should ensure access to individual income security as well as minimize the financial burden of medical costs. It is well documented that a health shock (i.e., a catastrophic medical expense) can lead to poverty [20–23]. The process of economic poverty caused by a disease or worsening of health status involves direct losses (i.e., medical expenditures) as well as indirect losses (i.e., income loss). Therefore, this study first investigated the effect of hospitalization on income loss and the differences in the effects by gender and employment status among South Korean workers. Second, it redefined the concept and meaning of the UHC’s definition in healthcare and social protection systems in South Korea based on the findings. This study reveals the importance of measures that ensure the right to work and the right to health among workers who are vulnerable to poverty caused by a health shock.

It was identified that income loss was greater for non-standard and self-employed workers than for standard workers, when hospitalized. It should be noted that while income loss was seen in groups of non-standard working women and self-employed men when the length of hospitalization was 7 days or less, a statistically significant income loss was also observed among non-standard working males when the stay was extended beyond 14 days. Previous studies also revealed a greater income loss among workers who experienced serious diseases such as cancer, especially those who were non-standard workers or low-income earners [7, 24]. Meanwhile, Sparrow et al. [25] reported a greater income loss due to worsening health among non-standard workers (-226.8 %) and the self-employed (-53.7 %), who were not in a low-income bracket [25]. A study in Korea found that non-standard workers who experienced a health shock are more likely to lose their jobs than standard workers [16]. The differential effect on income loss by employment status, even for those not belonging to low-income workers, may reflect differences in the type of work by employment status and availability of a workplace welfare system, such as a paid leave. Non-standard workers with precarious labor can experience unemployment when they are sick, without the ability to take a leave of absence to recover their health [26].

Especially among wage earners, income loss due to hospitalization occurred mainly among non-standard working females. These findings are consistent with previous studies that reported a greater income gap for women than for men when workers get sick, or that only women showed a loss of income [5, 27–29]. It is worth noting that, in this study, despite the average length of hospitalization by gender being longer for men than for women and the higher risk of male workers' disabilities, women's loss of income was greater. This can be seen from a labor-social context in Korea, which has a particularly high rate of non-standard work among women compared to men and the highest income gap among OECD member countries. According to the ratio of non-standard workers by gender in Korea, the proportion of non-standard workers among total wage earners is quite high at 45 % for women, compared with 29.4 % for men in 2019 [30]. The proportion of non-standard workers among female workers is higher than that of men, especially in the form of temporary and part-time employment with lower income. Moreover, the hourly wage gap between male and female workers is 34.1 %, the largest among OECD countries [31]. Further, social insurance in South Korea, which includes only workers with standard employment arrangements, hardly protects the precariously employed. Therefore, it is expected that Korean women, particularly women with non-standard employment arrangements, are higher vulnerable to loss of income when they fall sick.

In contrast, income loss was significant only among self-employed men. Compared to men, Korean women do not easily work as wage workers, especially because they get older. During this time, when the spouse runs a business, women work as unpaid family workers and support their husbands' work [32]. In this study, this possibility cannot be excluded because unpaid family workers without income were also included in the self-employed. Alternatively, not only wage workers, including regular and non-regular workers, but also self-employed women have a low average income of female groups such as unpaid family workers, which can lead to a flat effect of relatively small differences in income loss [13].

In addition to gender and employment status, this study confirmed that workers' income loss is affected by general characteristics. Middle-aged people from 45-65, who were engaged in economic activities, had relatively less income loss even if they were hospitalized, but elderly workers aged 65 or older lost a greater amount of income when hospitalized. In other words, elderly workers aged 65 years or older are much vulnerable to income loss when they fall sick [16]. Regarding marital status, male workers lost less income when they had a spouse than when they were single or widowed. This may be a phenomenon (a presentation), where in people endure and earn income even if they are sick due to responsibility for their families. Contrary to the results of previous studies, income loss occurred less when workers with spouses experienced hospitalization, which can be understood because this study did not distinguish between spouses' working status [16]. In contrast, it was insightful that women had opposite results, especially among divorced women, where they lived alone less than when they had a spouse. This can be due to the burden of childcare after divorce, or the economic burden of making a living alone. In conclusion, from a different perspective, both the large and small groups are likely to be at risk, and the group whose income loss was greater due to disease means they were at financial risk. In contrast, among the groups with relatively less income loss, it is predicted that men will continue to work with greater presenteeism, and women, for they either have childcare, or livelihood burdens. Therefore, if a social safety net such as paid sick leave or sickness allowance is established, it will not only support income loss, but also prevent workers' aforementioned presentation (working even when sick) and have a positive effect on family care in the long term. However, in the case of workers without private insurance or for the poor, the income loss get more severe.

Based on our findings, blind spots have been identified in the social security system for workers in the Korean labor market, who are in a complex crisis involving both health and unemployment. While many OECD countries have sickness benefits systems to ensure that workers take a fully rest during illnesses and recover from disease, such benefits are not available in South Korea, except for public officials. Several studies have reported positive effects of sickness benefits or paid sick leave that mitigate workers’ income loss, allowing them to take a rest, and, consequently, improve labor productivity [27, 33, 34]. Such benefits also allow the provision of timely treatment that in turn contributes to the maintenance and improvement of workers’ physical and mental health [35–37]. Given the current lack of a system to protect Korean workers from income loss (e.g., sickness benefits, paid sick leave) it is imperative to consider introducing and implementing the relevant system. Since non-standard workers and the self-employed are especially vulnerable to income loss, as they have no annual or monthly leaves at workplaces, it is critical to take a step-wise approach by setting priorities among groups while introducing the system. Australia and New Zealand have sickness benefits for the low-income group, and Germany implemented sickness benefits for the low-income group during the initial phase of introduction, followed by expansion of coverage in a step-wise manner. Switzerland operates and manages a separate sickness benefit system for the self-employed.

Based on the results of this study, an extended version of the WHO definition of the UHC may be proposed. As mentioned earlier, the meaning of UHC is to maintain and improve individuals’ health and address health inequality; therefore, it should be able to move beyond just medical costs and include the costs that can arise indirectly. Despite the widespread implications of the UHC concept, many countries have designed their UHC to cover only direct medical costs. Economic costs must be considered in addition to medical costs, and the demographic characteristics and vulnerability of various groups should be taken into account from a social perspective. With the onset of the COVID-19 pandemic, workers who have to be worriedy about their livelihoods are pointing out that it is difficult to take a rest in reality, paying attention to the role of social protection such as "sickness benefits." While infectious diseases spreads, such as COVID-19, sickness benefits have the following two key functions: 1) to ensure workers' right to take a rest, and 2) to prevent social problems such as the spread of infectious diseases. For example, tuberculosis (TB), a typical infectious disease with a long treatment period, increases the risk of poor TB treatment outcomes, exacerbates poverty, and contributes to sustaining TB transmission. Thus, it was noted that social protective interventions that prevent or mitigate other financial risks associated with TB, including income losses and non-medical expenditures such as transportation and food, are also important [38].

**Fig. 3:**
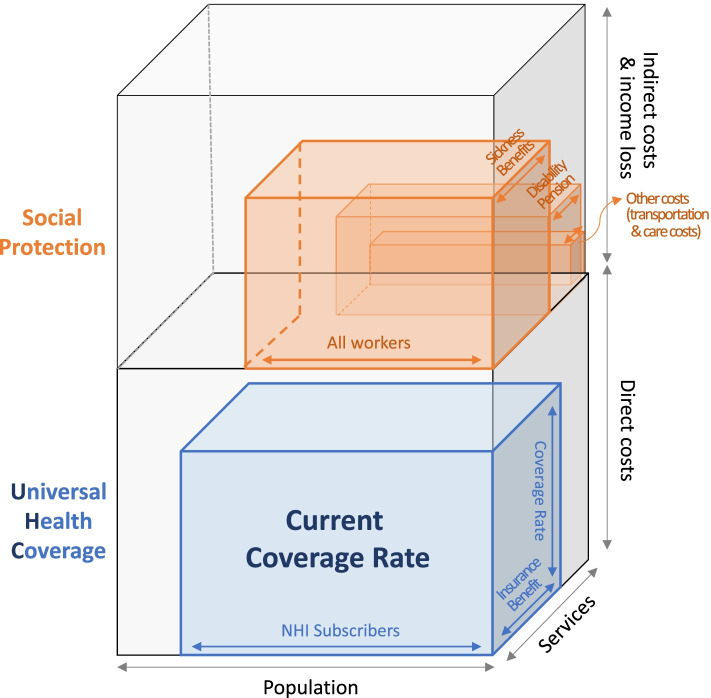
Universal health coverage (UHC) double cube model

Figure 3 shows the UHC double cube model presented by the authors, in which the blue cube shows the coverage of direct costs for existing health care expenditures, and the orange cube shows the coverage of indirect costs for social protection. In the blue cube, the three aspects describe who gets covered, what services are covered, and how much coverage those services receive from the WHO [2]. It is suggested that the orange cube consists of three smaller cubes with an extended concept. The first is the income loss cost arising from diseases of workers. Specifically, it is classified by the entity that compensates for the loss of income. For example, “paid sick leave” in which the user pays for some of the lost income and the workplace compensates for the rest, and the “sickness benefits” that compensate for workers’ income loss in the country. The second cube is the case where it is found permanently unearned, even though it is supported for ongoing medical expenses and income after the outbreak of the disease, and is a part that can primarily be guaranteed through the payment of a “disability pension” in social security pensions. The last cube indicates transportation costs, care costs, and other expenses that are not directly related to medical care. The extended concept of the UHC could take a step closer to bridging the health gaps that arise across the entire healthcare industry, as a system that can realize both universality and equity.

### Limitations

This study had several limitations. First, the disease severity was not considered. The KoWePS did not provide information on medical expenses or the diagnosis of diseases. However, as the data differentiated the length of hospital stay into short -and long-term, the length of hospitalization was used as a proxy for disease severity. Second, the study did not consider moderating factors such as private health insurance, social support, or use of non-income properties that a worker may use while being sick, except for the private health insurance used as a control factor in this study. Nevertheless, it is true in Korea that the greatest support during a worker’s sickness comes from a workplace support system or private insurance. Finally, there was a limitation to conduct more robust comparison across different types of workers and gender by performing stratification analysis rather than interaction analysis between hospitalization experience and work type or gender. Thus, in future studies, it is necessary to verify a clear causal relationship between income loss and the interaction terms of work type, gender, and hospitalization experience using sufficient number of samples.

## Conclusion

It is meaningful that this study confirmed indirect costs for loss of income through empirical analysis and proposed an extended concept of the UHC, as well as showing that support for workers’ access to medical care and medical expenses needs to be protected. As a results of this study, it was confirmed that if a worker is hospitalized for more than 2 weeks, it can affect income loss until the following year, as shown in prior studies that observed a long-term effect of loss of income [6, 12, 13]. Therefore, based on the present findings, it is reasonable to propose an alternative plan to secure at least a two-week service-guarantee period as part of developing Korea's paid sick leave or sickness benefit system. Several OECD countries benefit from at least two weeks of payment for sickness benefits [39]. Furthermore, it was confirmed that it is urgent to establish a system to protect all workers, including the vulnerable workers, considering there are extreme disparities in the labor market based on employment arrangements and gender. Based on the present findings, it is believed that introducing a paid sick leave or sickness benefits system for the entire population, including non-standard workers, self-employed workers, and standard women, will be necessary to implement the UHC. Thus, it is critical for the Korean government to continuously intervene and undertake efforts to ensure universal health protection to fight the resulting poverty, and address the income and health gaps.

## Supplementary Information

**Additional file 1. **

## Data Availability

The data that support the findings of this study are available from ‘the Korea Welfare Panel Study (KoWePS), 2009-2016’ conducted by the Korea Institute for Health and Social Affairs (KIHASA) at https://www.koweps.re.kr:442/main.do.

## Abbreviations

UHC: Universal Health Coverage
WHO: World Health Organization
OOP: Out-of-pocket
ILO: International Labor Organization
OECD: Organization for Economic Co-operation and Development
KoWePS: Korean Welfare Panel Study
SES: Socio-economic status
ROC: Receiver operating characteristics
NHI: National Health Insurance
