# Supplementary material for: Who loses more? Identifying the relationship between hospitalization and income loss: prediction of hospitalization duration and differences of gender and employment status
Source: BMC Public Health. 2022 Feb 4;22:232. doi: 10.1186/s12889-022-12647-6 (PMC8814781; doi:10.1186/s12889-022-12647-6)
Supplement: Supplementary file 1 — Additional file 1. [file 12889_2022_12647_MOESM1_ESM.docx]

**Appendix 1**. The effect of hospitalization more than 14 days on earned income loss (t_0_) by gender among Korean workers, 2011-2016

|  | | Men | | | | | | | | | Women | | | | | | | | |
| --- | --- | --- | --- | --- | --- | --- | --- | --- | --- | --- | --- | --- | --- | --- | --- | --- | --- | --- | --- |
|  |  | Regular (N=1,221) | | | Non-regular (N=706) | | | Self-employed (N=938) | | | Regular (N=556) | | | Non-regular (N=986) | | | Self-employed (N=469) | | |
|  |  | β | SE | p | β | SE | p | β | SE | p | β | SE | p | β | SE | p | β | SE | p |
| Age (ref: 18 ≤ age < 45) | |  |  |  |  |  |  |  |  |  |  |  |  |  |  |  |  |  |  |
|  | 45 ≤ age < 65 | **934.73** | **147.83** | **<0.001** | **-382.11** | **132.95** | **0.004** | -422.56 | 280.93 | 0.133 | **1066.99** | **190.21** | **<0.001** | **220.52** | **84.71** | **0.009** | 300.04 | 198.04 | 0.130 |
|  | age ≥ 65 | **-1043.88** | **363.86** | **0.004** | **-1540.08** | **165.14** | **<0.001** | **-1691.73** | **302.55** | **<0.001** | 843.22 | 634.18 | 0.184 | -124.91 | 115.54 | 0.280 | -34.87 | 227.39 | 0.878 |
| Education (ref: Under elementary school) | |  |  |  |  |  |  |  |  |  |  |  |  |  |  |  |  |  |  |
|  | middle school | -83.94 | 532.66 | 0.875 | 43.93 | 157.20 | 0.780 | -89.56 | 146.48 | 0.541 | 481.19 | 599.58 | 0.422 | 35.93 | 92.49 | 0.698 | -36.13 | 124.05 | 0.771 |
|  | high school | **964.24** | **441.83** | **0.029** | 228.23 | 140.30 | 0.104 | 134.23 | 140.26 | 0.339 | **1903.15** | **563.22** | **0.001** | 171.12 | 91.58 | 0.062 | **407.47** | **140.69** | **0.004** |
|  | college or higher | **1852.45** | **443.04** | **<0.001** | 232.95 | 165.52 | 0.159 | -238.88 | 191.77 | 0.213 | **3021.00** | **567.28** | **<0.001** | **311.69** | **107.74** | **0.004** | **601.69** | **197.18** | **0.002** |
| Marital status (ref: married couple) | |  |  |  |  |  |  |  |  |  |  |  |  |  |  |  |  |  |  |
|  | widowed | **-1653.65** | **721.88** | **0.022** | -128.72 | 268.80 | 0.632 | **-926.09** | **257.14** | **<0.001** | 554.37 | 500.01 | 0.268 | 86.91 | 92.49 | 0.698 | 82.73 | 90.71 | 0.362 |
|  | divorced or separated | -345.59 | 337.18 | 0.305 | -159.65 | 148.15 | 0.281 | -114.67 | 252.26 | 0.649 | -21.09 | 369.13 | 0.954 | **260.15** | **84.05** | **0.002** | **478.59** | **186.74** | **0.010** |
|  | single | **-1181.82** | **196.24** | **<0.001** | **-1062.16** | **131.12** | **<0.001** | **-1056.88** | **334.81** | **0.002** | -53.07 | 175.34 | 0.762 | 162.12 | 89.51 | 0.070 | 116.99 | 266.70 | 0.661 |
| National health insurance (ref: Yes) | |  |  |  |  |  |  |  |  |  |  |  |  |  |  |  |  |  |  |
|  | Medical aids | -371.62 | 1068.28 | 0.728 | **-599.16** | **226.52** | **0.008** | -604.26 | 491.37 | 0.219 | -480.57 | 1012.18 | 0.635 | -55.56 | 107.02 | 0.604 | -241.87 | 227.89 | 0.289 |
| Private health insurance (ref: No) | |  |  |  |  |  |  |  |  |  |  |  |  |  |  |  |  |  |  |
|  | Yes (≥1) | 199.63 | 131.78 | 0.130 | 69.77 | 80.79 | 0.388 | **322.33** | **109.51** | **0.003** | 107.55 | 172.71 | 0.533 | 94.45 | 50.03 | 0.059 | **196.97** | **78.54** | **0.012** |
| Poverty (ref: No) | |  |  |  |  |  |  |  |  |  |  |  |  |  |  |  |  |  |  |
|  | Yes | **-2322.21** | **270.94** | **<0.001** | **-858.02** | **94.71** | **<0.001** | **-1261.46** | **100.94** | **<0.001** | -905.08 | 500.01 | 0.268 | **-496.89** | **54.60** | **<0.001** | **-417.98** | **75.03** | **<0.001** |
| Chronic disease (ref: No) | |  |  |  |  |  |  |  |  |  |  |  |  |  |  |  |  |  |  |
|  | Yes | 68.69 | 108.29 | 0.526 | 109.87 | 83.01 | 0.186 | 65.42 | 106.52 | 0.539 | 74.46 | 133.97 | 0.578 | -27.71 | 44.77 | 0.536 | -151.33 | 88.44 | 0.087 |
| Disability (ref: No) | |  |  |  |  |  |  |  |  |  |  |  |  |  |  |  |  |  |  |
|  | Yes | -136.30 | 316.02 | 0.666 | **-288.71** | **144.42** | **0.046** | -247.39 | 144.96 | 0.088 | -258.97 | 739.22 | 0.726 | -147.13 | 156.04 | 0.346 | -27.46 | 124.34 | 0.825 |
| Subjective health status (ref: Very good) | |  |  |  |  |  |  |  |  |  |  |  |  |  |  |  |  |  |  |
|  | Good | 14.19 | 107.67 | 0.895 | 43.27 | 102.73 | 0.674 | -153.29 | 167.53 | 0.360 | **251.18** | **126.18** | **0.047** | -33.15 | 56.61 | 0.558 | 225.02 | 139.87 | 0.108 |
|  | Fair | **-330.91** | **166.22** | **0.047** | -72.22 | 128.07 | 0.573 | -121.04 | 186.55 | 0.516 | **414.01** | **203.48** | **0.042** | -39.41 | 68.10 | 0.563 | 213.61 | 154.97 | 0.168 |
|  | Bad | **-868.08** | **166.13** | **0.003** | -176.25 | 154.33 | 0.253 | -272.67 | 202.64 | 0.178 | -169.80 | 343.69 | 0.621 | -9.79 | 82.13 | 0.905 | 107.49 | 160.49 | 0.503 |
|  | Very bad | 1007.07 | 934.58 | 0.281 | -377.23 | 365.73 | 0.302 | -467.62 | 398.54 | 0.241 | 125.12 | 1369.16 | 0.927 | -48.44 | 235.29 | 0.837 | -290.25 | 265.16 | 0.274 |
| Hospitalization (≥14days) | |  |  |  |  |  |  |  |  |  |  |  |  |  |  |  |  |  |  |
|  | t_0+1_ | -312.17 | 313.98 | 0.32 | -266.21 | 166.32 | 0.109 | **-543.15** | **160.87** | **0.001** | -244.9 | 383.54 | 0.523 | **-320.11** | **91.07** | **<.001** | -72.05 | 98.03 | 0.462 |
